# Discovery, Biosynthesis, and Characterization of Rodencin, a Two-Component Lanthipeptide, Harboring d-Amino Acids Introduced by the Unusual Dehydrogenase RodJ_A_

**DOI:** 10.1021/acs.jnatprod.4c00170

**Published:** 2024-09-20

**Authors:** Yuxin Fu, Eleftheria Pateri, Oscar P. Kuipers

**Affiliations:** † Department of Molecular Genetics, Groningen Biomolecular Sciences and Biotechnology Institute, University of Groningen, Groningen 9747 AG The Netherlands

## Abstract

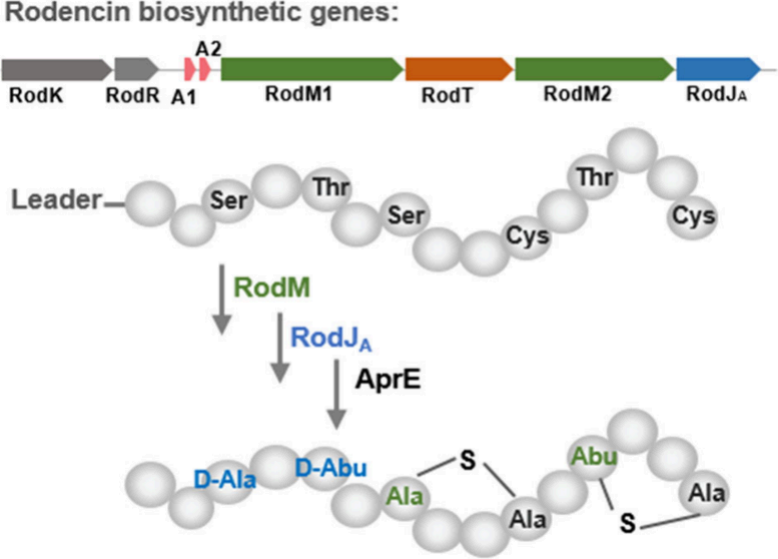

Lanthipeptides, a group of ribosomally synthesized and post-translationally modified peptides (RiPPs), exhibit diverse structures and bioactivities. Their biosynthetic enzymes serve as valuable tools for peptide bioengineering. Here, we report a class II lanthipeptide biosynthetic gene cluster in a *Bacillus* strain, driving the biosynthesis of a two-component lanthipeptide, termed rodencin, featured by the presence of two different d-amino acids, i.e., d-Ala and d-Abu. Rodencin displays synergistic antimicrobial activity against food-borne pathogens such as *Bacillus cereus*, *Staphylococcus aureus*, and *Listeria monocytogenes*. The α-peptide of rodencin contains one d-Ala and the β-peptide features both d-Ala and d-Abu. These are installed by dehydratases RodM1 and RodM2 and dehydrogenase RodJ_A_, the activities of which were successfully reconstituted using a dedicated *E. coli* expression system. To illustrate the unusual d-Abu incorporation potential of the enzymes, analogous to the d-amino acid-containing β peptide of lacticin 3147, was successfully produced with the rodencin heterologous expression system, by employing RodM2 and the dehydrogenase RodJ_A_.

Lanthipeptides, extensively studied within the category of ribosomally synthesized and post-translationally modified peptides (RiPPs), are widespread among diverse bacterial species.^1,2^ Many lanthipeptides demonstrate potent antimicrobial properties and these are termed lantibiotics.^3,4^ Structure–activity relationship studies have revealed the crucial bioactivity brought about by their characteristic cross-linked thioether structures, known as lanthionine (Lan; Ala-S-Ala) and/or methyllanthionine (MeLan; Abu-S-Ala or Ala-S-Abu).^5,6^ These (Me)Lan moieties also confer rigidity, proteolytic stability, and target specificity^7−9^ by constraining the conformation of these peptides. They are formed through dehydration of serine (Ser) and threonine (Thr) residues, followed by an intramolecular Michael-type addition of cysteine (Cys) thiols to the resulting dehydroalanine (Dha) or dehydrobutyrine (Dhb) residues.^5^ Post-translational modifications (PTMs) are exclusively introduced to the C-terminal core peptide, while the N-terminal leader sequence serves as a recognition and binding site for modification enzymes and the ABC transporter (LanT). In the final maturation step, a LanP protease proteolytically removes the leader sequence.^5^ Further classification has revealed five distinct classes (I–V) of lanthipeptides based on variations in biosynthetic enzymes for (Me)Lan ring installation.^10,11^ In class I lanthipeptides, the dehydration and cyclization processes are executed by separate enzymes, namely, LanB and LanC, while for class II lanthipeptides, a singular bifunctional enzyme, LanM, catalyzes both reactions (Figure 1). Class II lanthipeptides can be single-component or two-component systems. Most two-component lanthipeptides feature a lipid II-binding α-peptide working synergistically with a β-peptide, resulting in significantly enhanced bioactivity. (Me)Lan ring installation of two-component lanthipeptides may be facilitated by a single LanM (e.g., carnolysin and bicereucin)^12,13^ or by two distinct LanMs, each specific to α or β precursor peptides (e.g., lacticin 3147, haloduracin).^14,15^ Notably, certain LanBCs and LanMs, such as NisBC from the nisin biosynthetic gene cluster and ProcM and SyncM from marine cyanobacteria, provide well-established tools for lanthipeptide engineering, as demonstrated in numerous examples of lanthionine ring introduction.^16−18^

**Figure 1:**
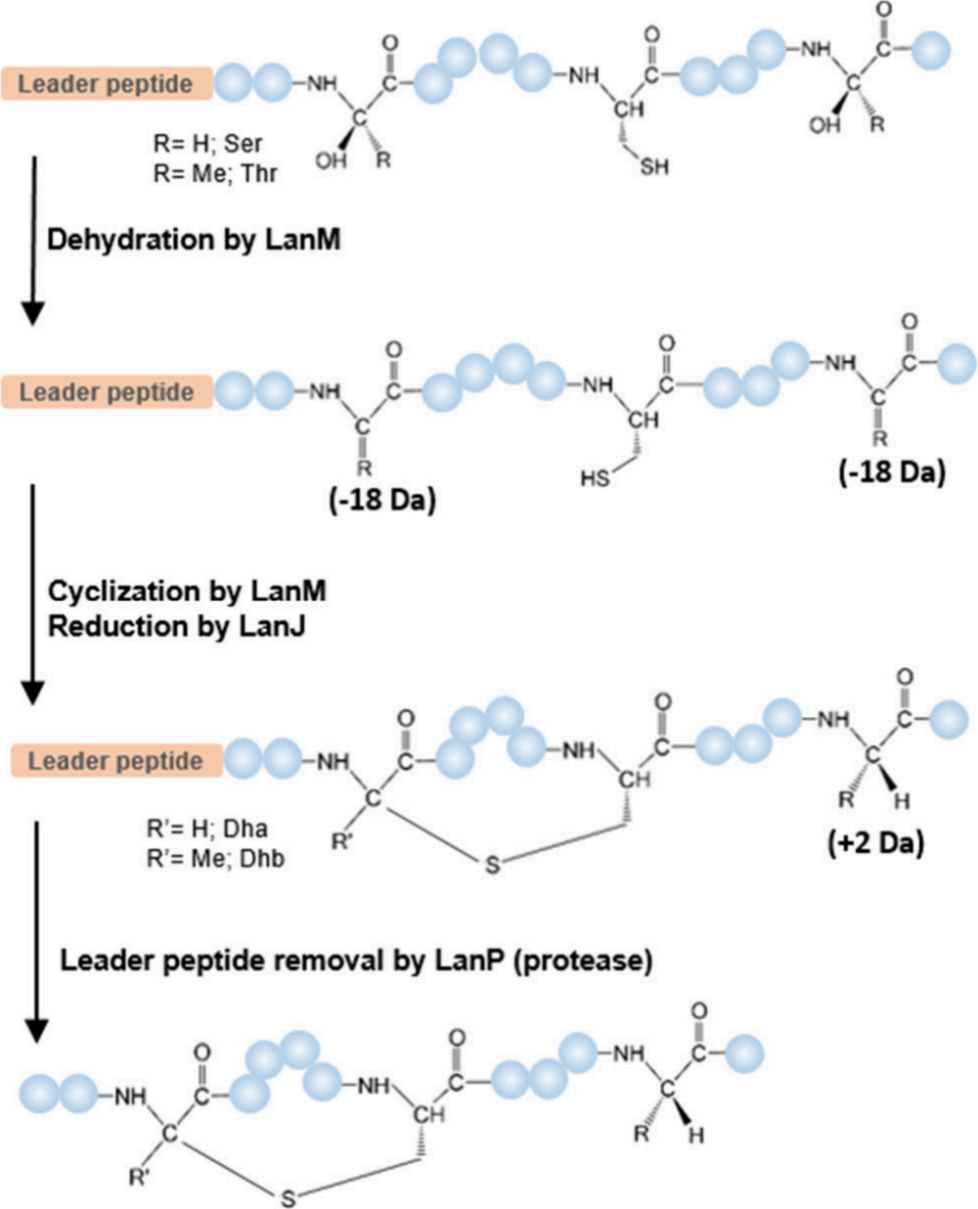
Schematic presentation of the biosynthesis of d-amino acid containing lanthipeptide. Bifunctional (dehydration and cyclization) enzyme, LanM, introduces dehydrated residues and (Me) Lan rings into precursor peptides; dehydrogenase LanJ facilitates the reduction of dehydrated residues Dha and/or Dhb to d-Ala and/or d-Abu, and the leader peptides of precursor peptides are proteolytically removed by a LanP or other protease in the final step of maturation.

Besides (methyl)lanthionine, the comprehensive exploration of PTMs in lanthipeptides, has been achieved with the advent of genome mining. Previous studies have thoroughly discussed the richness and complexity of PTMs in lanthipeptides, underscoring the pivotal role of biosynthesis enzymes and their potential for engineering.^5,19^d-Amino acids, being nonproteinogenic residues, confer various advantageous properties to peptides, including enhanced bioactivity, increased resistance to proteolysis, and stereochemical constraints for downstream biosynthesis into the final products.^20,21^ Given that the ribosomal machinery favors only l-amino acids, employing PTM methods to introduce d-stereocenters into peptides becomes valuable. A small subset of lanthipeptides contain d-amino acids introduced through two enzymatic reactions: the dehydratase domain of a lanthionine synthetase catalyzes the initial process, followed by the reduction of Dha and/or Dhb residues to d-Ala and/or d-amino butyric acid (d-Abu), facilitated by a dehydrogenase termed LanJ (Figure 1).^1,22^ The LanJ-mediated introduction of d-amino acids is observed to be limited, primarily occurring in class II lanthipeptides and a recently discovered class V lanthipeptide.^23^ These enzymes can be classified into three distinct dehydrogenase categories: the zinc and NADPH-dependent dehydrogenases (LanJ_A_), such as the LtnJ_A_ involved in the biosynthesis of lacticin 3147,^24^ flavin-dependent dehydrogenases (LanJ_B_), such as BsjJ_B_ involved in the biosynthesis of bicereucin,^12^ and F420H2-dependent reductases (LanJ_C_) recently found in class V lanthipeptides.^23^ Unlike the LanJ_B_ class of enzymes capable of reducing both Dha and Dhb, the characterized LanJ_A_ enzymes exhibit a more confined function, reducing only Dha to d-Ala.^5^ Previous engineering explorations of LanJ enzymes have primarily focused on the d-Ala incorporation capability, with NpnJ_A_ and LtnJ_A_ being the subjects of investigation.^22,25^ However, the detailed exploration of LanJ_B_’s engineering potential in terms of d-Abu incorporation has remained unexplored. In this study, we identified a novel dehydrogenase, RodJ_A_, as the first reported LanJ_A_ enzyme capable of reducing both Dha and Dhb. We reconstituted the activity of this RodJ_A_ enzyme in heterologous expression systems, exploring its engineering potential to incorporate d-Abu into non-native substrates.

Here, we report a structural and biosynthetic investigation of a d-amino acid containing two-component class II lanthipeptide, named rodencin. Rodencin is derived from *Bacillus subtilis* EH5, an isolate from tomato rhizosphere soil, characterized by the inclusion of d-Ala in the α-peptide and of both d-Ala and d-Abu in the β-peptide. The heterologous expression system of rodencin presented is functional and was applied to produce active rodencin. Biosynthetic investigations revealed that the formation of d-Ala and d-Abu relies on putative NADPH-dependent dehydrogenase RodJ_A_, sharing high sequence similarity with LanJ_A_ enzymes of known lanthipeptide biosynthetic pathways. Furthermore, we present evidence of unprecedented incorporation of d-Abu into unnatural products using RodJ_A_, showcasing the feasibility of using the unique biosynthetic enzymes of rodencin for lanthipeptide engineering. These findings demonstrate the previously unrecognized function of LanJ_A_ enzymes to introduce d-Abu and underscore the potential of rodencin biosynthetic enzymes in expanding the structural diversity of the peptides.

## Results and Discussion

### Description of Novel Gene Clusters from *Bacillus subtilis* EH5

**Figure 2:**
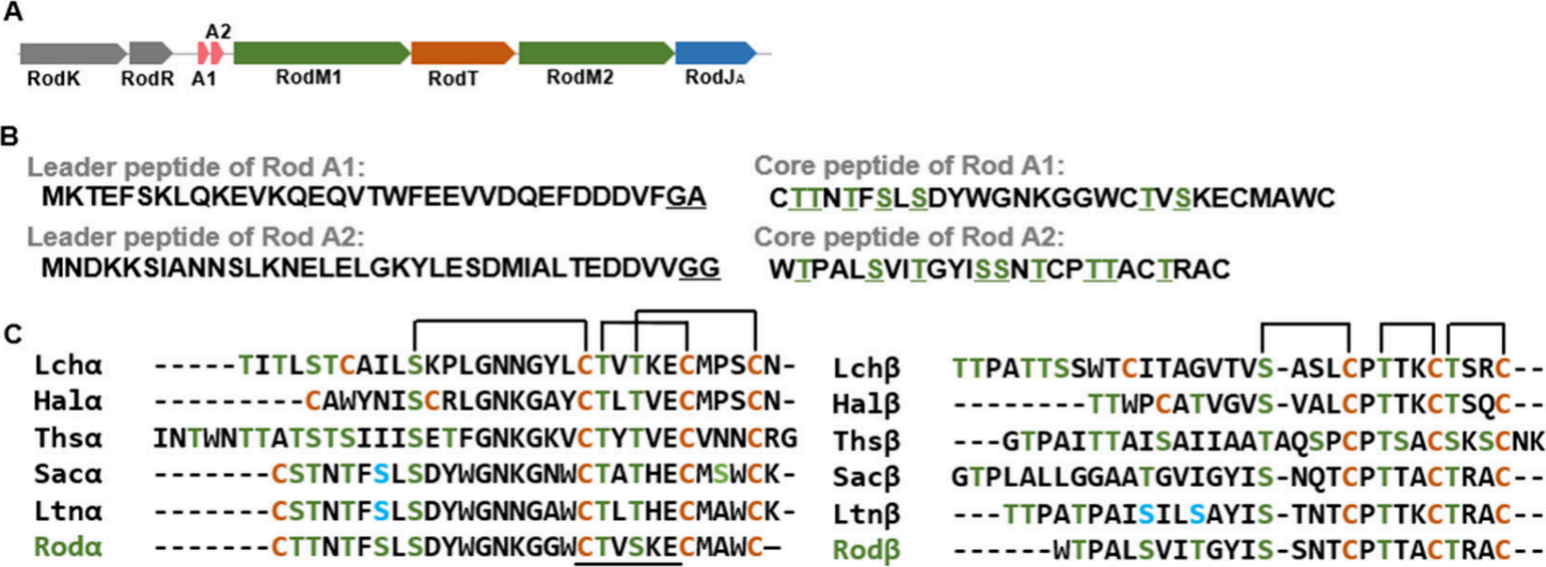
(A) The biosynthetic gene cluster for rodencin. (B) The amino acid sequence of precursor peptide of rodencin and the predicted cleavage site. (C) Sequence alignment of core peptides of selected two-component lanthipeptides with that of rodencin, and representative ring structures of the peptides are indicated. Cys residues are marked in orange. Ser/Thr residues that are dehydrated are shown in green. Ser/Thr residues, post-translationally modified to d-Ala/Abu, are shown in blue. The conserved lipid II binding motif CTxT/SxEC, typically found in the α-component of most two-component lanthipeptides, is underlined. d-Amino acids in rodencin are not indicated.

*Bacillus subtilis* EH5 is an antimicrobial substance-producing strain which was isolated during the screening of potential plant growth-promoting rhizobateria from tomato rhizosphere soil.^26^ Genome mining revealed the presence of a novel two-component class II lanthipepitde biosynthetic gene cluster (BGC) in *B. subtilis* EH5, which we designate rodencin. This gene cluster contains short open reading frames for the two precursor peptides RodA1 and RodA2 (Figure 2A). Like most lanthipeptides, these peptides appear to have N-terminal leader peptides consisting of 35 and 37 amino acids, respectively. The leader peptide termini were predicted by the presence of a typical double-glycine and glycine-alanine motif, respectively (Figure 2B).^27,28^ A search of the nonredundant protein sequence database for homologues of the C-terminal core peptide (Rodα and Rodβ) of precursor peptides showed their similarity to the d-alanine-containing two-component lanthipeptides lacticin 3147 and staphylococcin C55. RodA1 has a core peptide sequence nearly identical to those of LtnA1 and SacA1 but without a lysine at the C-terminus. RodA2 has a shorter but similar core peptide as compared to that of LtnA2 and SacA2 (Figure 2C). The gene cluster also encodes two putative class II lanthipeptide synthetases RodM1 and RodM2, a peptidase-containing ATP-binding cassette transporter RodT, and a dehydrogenase^13,29^ RodJ_A_, a homologue of LanJ_A_ dehydrogenases present in lacticin 3147, and staphylococcin C55 gene clusters. Phylogenetic analysis of the retrieved sequences of known LanJ enzymes showed that RodJ_A_ forms a clade with the representative LanJ_A_ enzymes (Supporting Information (SI), Figure S1A). The alignment of the amino acid sequence of RodJ_A_ with other LanJ_A_ enzymes revealed that the residues conserved to be important for LtnJ_A_ function,^30^ and the protein domains conserved in LtnJ_A_-like proteins are also present in RodJ_A_. However, some interesting deviations were also observed in RodJ_A_, such as residues Arg^100^ and Asp^101^ within the conserved domain, as well as residues Met^112^ and Ser^113^, located near the presumed zinc ligand. These deviations may explain its wider substrate specificity (SI, Figure S1B,S1C).

### Detection of Rodencin Production

A single *B. subtilis* EH5 colony was inoculated in LB liquid medium and grown overnight. Subsequently, the culture was diluted 100 times into a minimum essential medium (MEM) for peptide production. MEM was used to reduce the background noise from the medium when detecting production of the putative lanthipeptides after 12, 24, and 48 h with matrix-assisted laser desorption/ionization time-of-flight (MALDI-TOF). A mass corresponding to the core peptide of RodA2 (Rodβ) with six dehydrations and two reductions was observed after 24 h (SI, Figure S2). However, the potential mass corresponding to the core peptide of RodA1 (Rodα) was not detected even after 48 h. This discrepancy may be attributed to differences between the experimental growth conditions employed and the natural environment.

### Biosynthesis of Rodencin in *Escherichia coli*

To characterize rodencin (Rodα and Rodβ) and its biosynthetic pathway, heterologous production of rodencin and rodencin variants in the standardized expression host *Escherichia coli* was conducted. The RodM1-J_A_ enzymes were coexpressed with their substrate precursor peptide RodA1, and RodM2-J_A_ were coexpressed with RodA2. Cross-pairing was also performed to verify whether the two synthases corresponded specifically to their precursor peptide. The genes encoding the precursor peptides were amplified from genomic DNA of *B. subtilis* EH5 and were fused to the His-tag sequence of the pCDFDuet-1 vector, resulting in histidine-tagged precursor peptides (His-RodA1 and His-RodA2). The coding regions for synthases RodM and dehydrogenase RodJ_A_ were amplified and placed in the multiple cloning site 1 (MCS-1) and MCS-2 of the pRSFDuet-1 vector, respectively. These constructs yielded untagged RodM1-J_A_ and RodM2-J_A_ enzyme complexes. *E. coli* BL21 (DE3) cells were transformed with both plasmids and after peptide expression, cells were broken by sonication, the lysate was subjected to centrifugation to discard the dense cell material and then purified by immobilized metal affinity chromatography (IMAC). The soluble expression products of His-RodA1 and His-RodA2 were demonstrated by tricine-SDS-PAGE as shown in SI, Figure S3A. The almost invisible production of RodM2-J_A_ modified His-RodA1 and RodM1-J_A_ modified His-RodA2 (SI, Figure S3B) demonstrates that the synthetase RodM1 is dedicated to RodA1 and RodM2 is specialized to modify RodA2.

**Figure 3:**
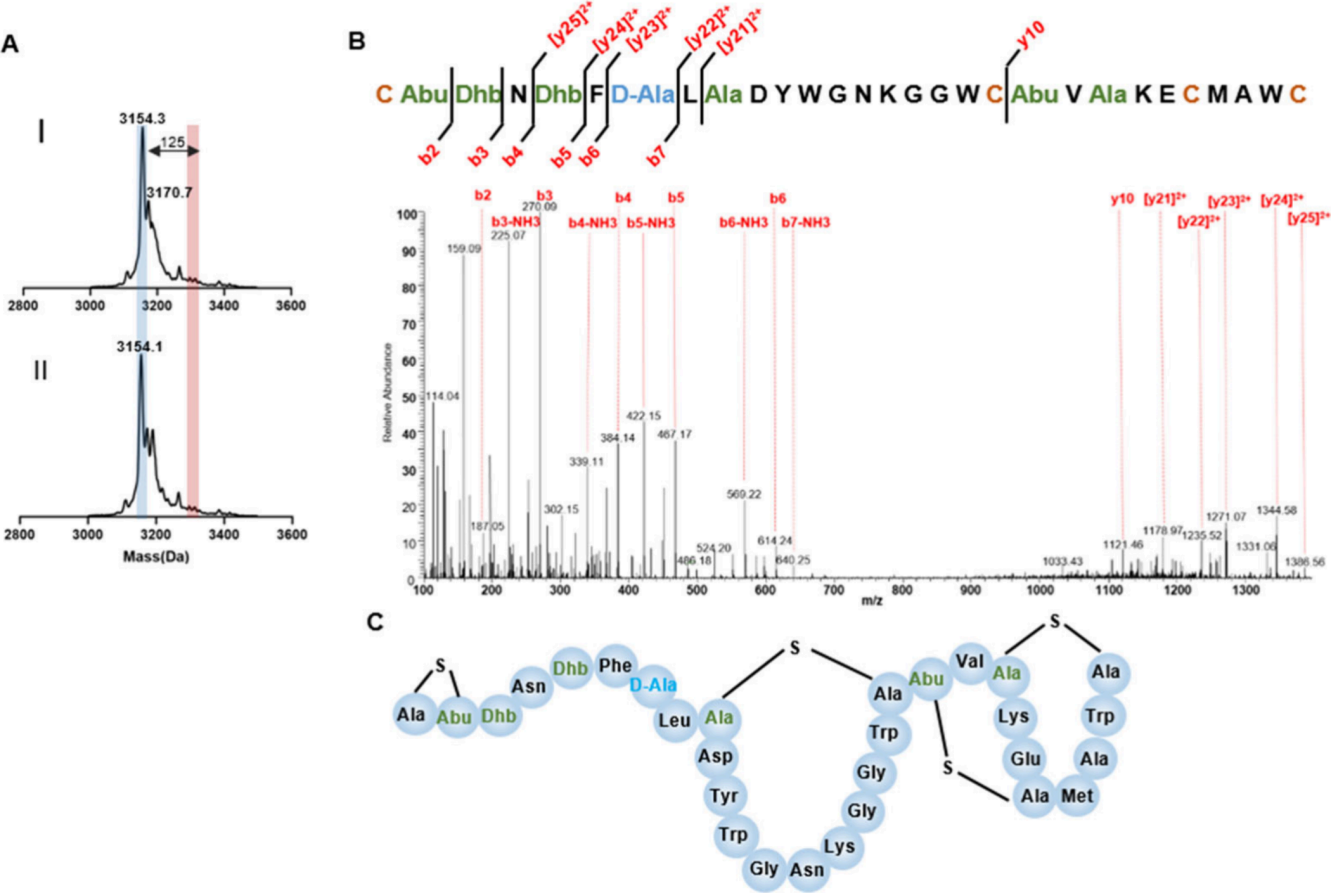
Posttranslational modification analysis of heterologously expressed Rodα. (A) MALDI-TOF for heterologous expressed Rodα before (I) and after (II) treatment with *N*-ethylmaleimide (NEM), a cysteine-reactive alkylating reagent. The NEM reaction causes a mass shift of 125 Da, indicated in red. (B) LC-MS/MS fragments pattern of heterologous expressed Rodα. (C) The structure of Rodα assigned in this work.

**Figure 4:**
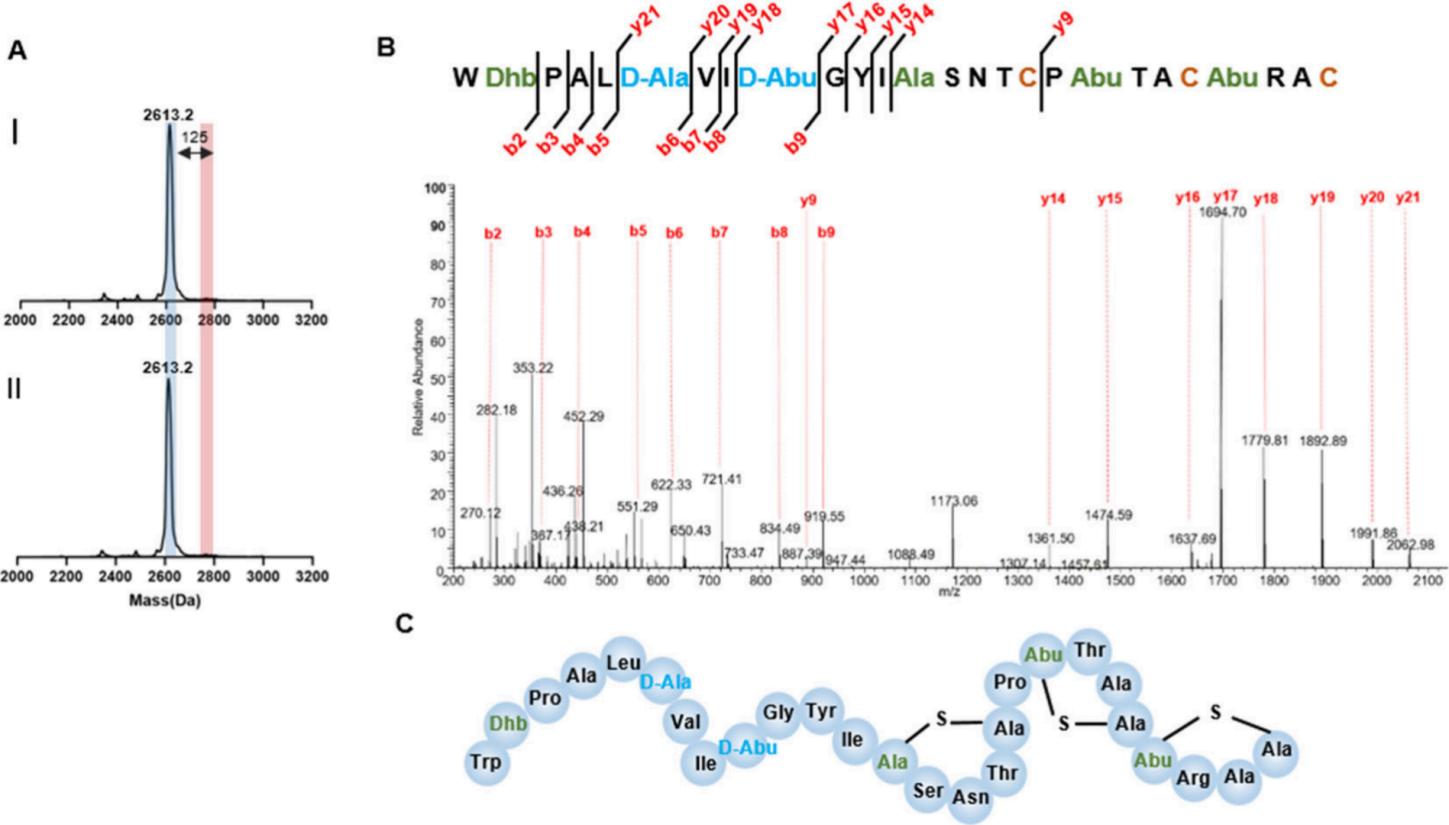
Posttranslational modification analysis of heterologously expressed Rodβ. (A) MALDI-TOF for heterologous expressed Rodβ before (I) and after (II) treatment with *N*-ethylmaleimide (NEM), a cysteine-reactive alkylating reagent. The NEM reaction causes a mass shift of 125 Da, indicated in red. (B) LC-MS/MS fragments pattern of heterologous expressed Rodβ. (C) The structure of Rodβ assigned in this work.

To determine the exact masses of the core peptides of the resulting products, the proteolytic removal of the leader peptide was investigated. Usually, leader peptides terminating in the GA/GG motif are removed by the cysteine protease domain of LanT transporters,^27^ and indeed RodT is present in the gene cluster. We discovered that mixing the heterologously expressed peptide products with supernatant of *B. subtilis* EH5 resulted in the detection of masses corresponding to modified RodA1 and RodA2 cleaved after the GG/GA sequence, suggesting the presence of an extracellular protease responsible for leader cleavage. Thus, 4352 annotated proteins produced by *B. subtilis* EH5 were inspected. One serine alkaline protease (subtilisin E, AprE) was searched as the only extracellular protease, having a sequence identical with that of AprE of *B. subtilis* 168. The readily available semipure *B. subtilis* 168 protease AprE^31^ was used to cleave RodM1-J_A_ modified His-RodA1 and RodM2-J_A_ modified His-RodA2 *in vitro*. The resulting products were analyzed by MALDI-TOF, and a mass corresponding to seven dehydrations and one reduction of the core peptide of RodA1 was observed in the mass spectrum (Figure 3A), as well as a mass corresponding to six dehydrations and two reductions of the core peptide of RodA2 was detected, which is consistent with mature Rodβ isolated from the *B. subtilis* EH5 supernatant (Figure 4A, and SI, Figure S2). These results demonstrate that RodJ_A_ can reduce a subset of the Dha and/or Dhb residues to Ala and/or Abu, respectively, with six dehydro amino acids in Rodα and four dehydro amino acids in Rodβ escaping reduction. This also confirmed that AprE is responsible for *in vitro* leader cleavage of modified RodA1 and RodA2. Notably, the use of semipure *B. subtilis* 168 protease AprE or commercial AprE contributed to the ease of use of this *E. coli* expression system, avoiding the production and purification of RodT. Further investigation into whether RodT is responsible for the leader cleavage of rodencin would offer further insights into the biosynthetic mechanism of rodencin.

His-RodA1 or His-RodA2 were also individually coexpressed with RodM1 and RodM2 in *E. coli* BL21 (DE3) to explore the effects of the absence of RodJ_A_ on the biosynthesis of rodencin. After IMAC purification, analysis by tricine-SDS-PAGE (SI, Figure S4A) showed a good production level. Subsequently, purified His-RodA1 and His-RodA2 underwent AprE treatment, and MALDI-TOF MS analysis revealed the main peak corresponding to incomplete dehydration of core peptides with no observed reductions (SI, Figure S4B). These observations indicate the importance of RodJ_A_ for the full modification and biosynthesis of rodencin.

### Structural Characterization of Rodencin

To determine how many (Me)Lan rings are formed in rodencin (Rodα and Rodβ) by intramolecular coupling of cysteines to the dehydro-amino acids, *N*-ethylmaleimide (NEM), an alkylating reagent that reacts with free-thiols of cysteine adding a mass of 125 Da, was first used to react with the purified Rodα and Rodβ peptides. The results showed that no mass shift happened after the NEM reaction in both peptide cases (Figures 3A and 4A) (positive control with 125 Da mass increase is given in SI, Figure S5), suggesting all cysteines in Rodα and Rodβ are occupied and probably involved in the formation of four (Me)Lan rings in Rodα and three (Me)Lan rings in Rodβ. This was corroborated with liquid chromatography–tandem mass spectrometry (LC-MS/MS) analysis. LC-MS/MS is a widely used tool to analyze the dehydration pattern and exact ring formation in the modified peptides.^17,32^ The fragment ion spectra of protonated peptides (purified from AprE-cleaved RodA1 and RodA2 give clues to the amino acid composition of the peptides, which are consistent with the sequences of Rodα and Rodβ observed in sequenced genomes (Figures 3B and 4B). Because the (Me)Lan ring prevents the fragmentation in the ring, the first two nonoverlapping rings of Rodα were determined (the positions seen in Figure 3C). However, the spectrum shows no fragmentation in the C-terminal of Rodα, which indicates the overlapping rings formed here. The characteristic overlapping-ring pattern and the conserved lipid II binding motif CTxT/SxEC can be found in the α-component of most two-component lantibiotics.^33,34^ Thus, the third and fourth ring pattern of Rodα was assumed the same as that of other α-peptides of two-component lantibiotics (Figure 2C). The three nonoverlapping rings that constitute the clear ring pattern of Rodβ was also determined (Figure 4B,C).

The LC-MS/MS results also illustrated the positions of dehydro-amino acids and that the Dha residues at position 7 in Rodα and Dha at position 6 in Rodβ were reduced to d-Ala (Figures 3B and 4B). Notably, Dhb at position 9 was reduced to d-Abu in Rodβ (Figure 4B). This is unlike the specificity of other LanJ_A_ dehydrogenases characterized to date, such as LtnJ_A_ in lactacystin 3147 and SacJ_A_ in staphylococcin C55 biosynthesis, which can only reduce Dha despite the presence of Dhb residues in their sequences. Previous engineering studies on LtnJ_A_ underscored the inability of Dhb to serve as a substrate for LtnJ_A_,^35^ elucidating the confines of its specificity. RodJ_A_ was shown as the first LanJ_A_ enzyme that can reduce both Dha and Dhb. The proposed structures of Rodα and Rodβ are shown in Figures 3C and 4C. Further clarification through NMR would provide additional insights and enhance our understanding of the 3D structure of rodencin.

### Activity Characterization of Rodencin

**Figure 5:**
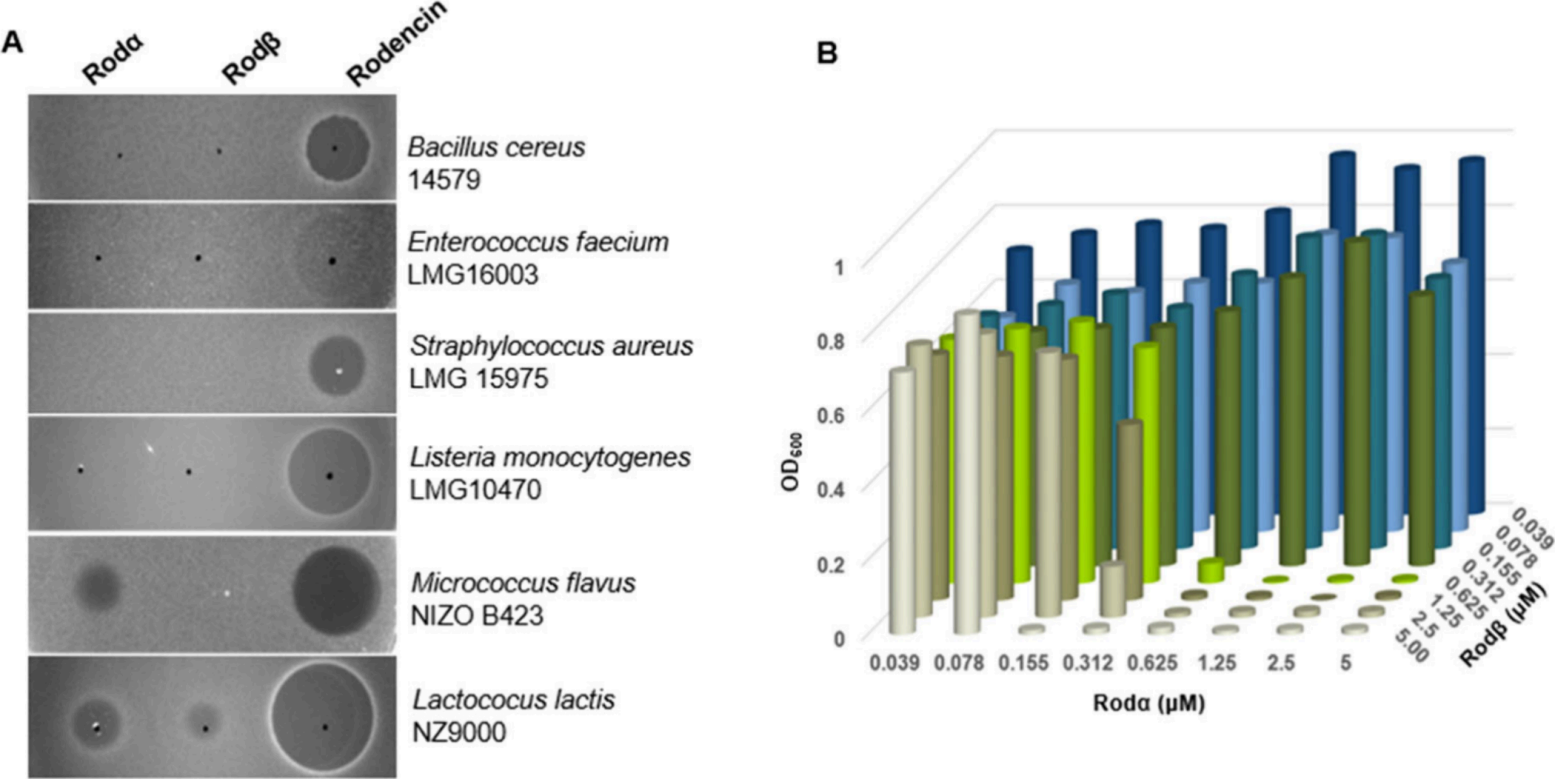
Antimicrobial activity assays of rodencin. (A) Agar diffusion growth assay, spotted total peptide amount: 0.2 nmol. (B) Isobologram demonstrating the optimal inhibitory ratio of Rodα and Rodβ, using *S. aureus* LMG15975 as indicator strain.

The biological activities of HPLC-purified Rodα and Rodβ were investigated with a range of bacteria as indicator strains. Neither Rodα nor Rodβ alone exhibited good activity against the tested pathogens. Only Rodα showed slight inhibition against *M. flavus* and *L. lactis*, while Rodβ alone exhibited slight inhibition against *L. lactis*. However, the Rodα and Rodβ mixtures had a dramaticly increased inhibition efficacy compared with the peptides used individually. Therefore, Rodα and Rodβ act synergistically to inhibit Gram-positive bacteria including the pathogens *B. cereus*, *L. monocytogenes*, and *E. faecium* and MRSA but not against any of the tested Gram-negative bacteria (Figure 5A). This result also confirms the functional expression of Rodα and Rodβ. Combined with the structural characterization of the AprE-cleaved peptides mentioned above, it verifies that AprE cleavage occurred at the correct site. The structures in Figures 3C and 4C represent native rodencin.

**Figure 6:**
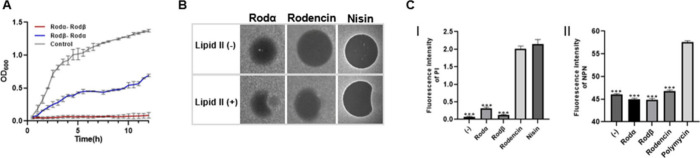
Antimicrobial properties of rodencin. (A) Sequential effects of Rodα and Rodβ on *S. aureus*. (B) Spot-on-lawn assay with *M. flavus* for investigating the lipid II-binding capacity of Rodα and rodencin. (C) Inner membrane permeability of the Gram-positive stain *M. flavus* under Rodα, Rodβ, and rodencin treatment (I), with 0.1 mg/L nisin used as positive control. Outer membrane permeability of the Gram-negative stain *E. coli* under Rodα, Rodβ, and rodencin treatment (II), with 0.5 mg/L polymyxin B used as positive control. The tests were performed in triplicate and the data are presented as mean ± SD; ***, *p* < 0.001 vs nisin or polymycin by Student’s *t* test.

Liquid growth inhibition assays were conducted to determine the optimal molar ratios of Rodα and Rodβ, and the resulting isobologram indicated that the ideal molar ratio between Rodα and Rodβ was found to be 1:1 (Figure 5B), and their corresponding minimal inhibitory concentrations (MICs) were found to be in the range of submicromolar concentrations (refer to SI, Table S1). To explore the potential binding order for inhibiting cell growth, we performed a previously reported sequential addition method of peptides (Figure 6A). Our findings are similar to those found in other two-component lanthipeptides, where Rodα binds to a target on the bacterial cell surface, forming a complex that is essential for the synergistic action of Rodβ.^36^ This was further confirmed through lipid II binding and membrane integrity assays. Lipid II is peptidoglycan precursor molecule, which is essential for bacteria cell wall synthesis^37^ and serve as the binding target of many lantibiotics, including nisin,^38^ mersacidin,^39^ and the α-peptide of two-component lantipeptides.^40−42^ The Rodα peptide has a lipid II binding motif, i.e., CTLTXEC, which is consistent with the general model of two-component lanthipeptides as underlined in Figure 2C, so we investigated the lipid II-binding capacity of Rodα and rodencin (mixture of Rodα and Rodβ) by an agar diffusion assay as described in previous studies.^43,44^ Due to binding to externally added purified lipid II, the antimicrobial activity of Rodα and rodencin against *M. flavus* was diminished, causing a disruption of the typically round antimicrobial halo (Figure 6B); this suggests that lipid II is a binding target for Rodα to form a complex. Nisin was used as a positive control for the lipid II-binding assay. Because rodencin exhibits activity against Gram-positive stains, we then measured the membrane permeability of the Gram-positive stain *M. flavus* under rodencin treatment using the DNA-binding dye propidium iodide (PI). The results showed that *M. flavus* exhibited uptake of PI by rodencin at MIC concentration, but not under treatment with only Rodα or Rodβ (Figure 6C). The outer membrane permeabilization of the Gram-negative strain to the hydrophobic fluorescent probe 1-*N*-phenylnaphthylamine (NPN) was also detected, with polymyxin B as a positive control. No outer membrane permeability of *E. coli* was observed after treatment with rodencin or the peptides alone (Figure 6C). Our results suggest that Rodα binds to lipid II on the peptidoglycan cell wall, which has also been indicated for other two-component lanthipeptides,^36,45^ and it facilitates Rodβ’s ability to synergistically permeate the membrane of Gram-positive bacteria. However, in Gram-negative strains, the peptidoglycan layer is enveloped by an outer membrane containing lipopolysaccharide,^46^ preventing rodencin from reaching lipid II. This proposition opens up possibilities for designing and engineering the α peptide to target alternative outer membranes or strain-specific targets, thereby guiding the β peptide to enhance permeability.

We also tested the activity of Rodα and Rodβ variants that were made without d-amino acids. First, we tested a combination of RodM1 individually modified Rodα and RodM2 individually modified Rodβ against *M. flavus*. This mixture demonstrated significantly decreased inhibitory efficacy compared with Rodα and Rodβ (SI, Figure S4C). Subsequently, we tested the activity of RodM1 individually modified Rodα with Rodβ and RodM2 individually modified Rodβ with Rodα. Both combinations exhibited decreased inhibitory efficacy compared to Rodα and Rodβ together, with the Rodβ variant lacking d-amino acids showing a particularly pronounced adverse effect on activity (SI, Figure S4C). We hypothesize that d-amino acids at the right position in the peptide backbones of antibiotics are important in the binding of these antibiotics to lipid II^47^ and penetrate the cell membrane.

### Lanthipeptide Engineering with the Rodencin Biosynthetic Machinery in *E. coli*

**Figure 7:**
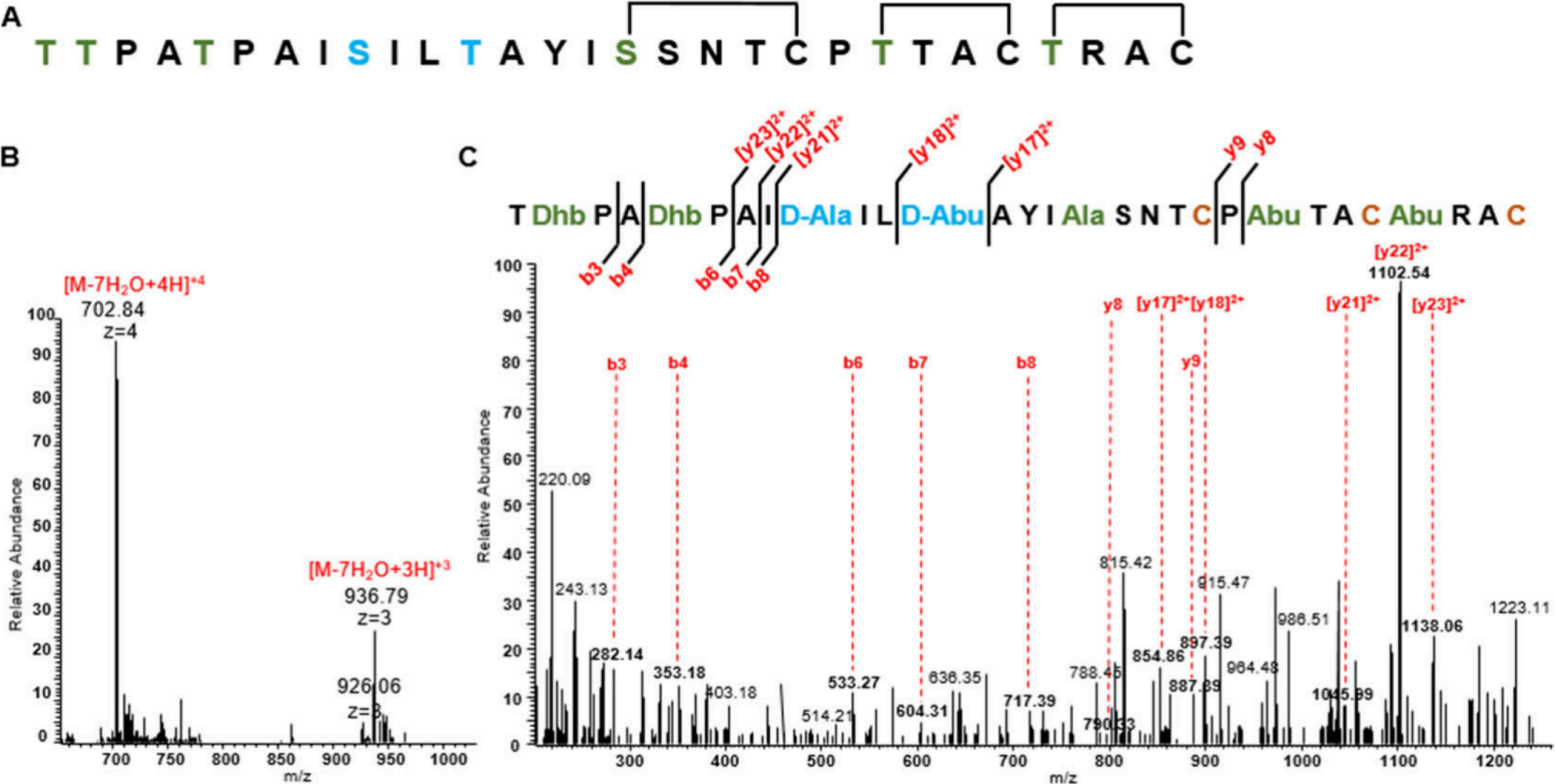
(A) The sequence of designed Ltnβ variant, the expected dehydrated amino acids are labeled in green, d-amino acid incorporation sites are labeled in blue, and the expected ring topology is indicated. (B) The masses of peptide ions found in LC-MS for RodM2-RodJ_A_ modified Ltnβ variant and the corresponding dehydration numbers. (C) LC-MS/MS fragments pattern of RodM2-RodJ_A_ modified Ltnβ variant with the rodencin modular expression system.

d-Amino acid introduction is highly valuable for peptide engineering, yet only a limited set of PTM enzymes have been reported for effectively introducing d-stereocenters into peptides for synthetic purposes. In this study, we explored the potential of the RodJ_A_ enzyme in preparing unnatural substrates containing d-amino acids with a particular emphasis on the insertion of d-Abu, an aspect that remains unexplored in peptide engineering. For this purpose, we utilized a designed Ltnβ variant, mirroring the C-terminal amino acid composition of Rodβ and featuring a substitution of the second Ser at position 12 from the N-terminal with Thr, as illustrated in Figure 7A. This variant served as the substrate for the RodM2-J_A_ enzymes in the rodencin modular expression system. The Ltnβ variant sequence was fused to the Rodβ leader on the plasmid. After coexpression with RodM2-J_A_ in *E. coli* BL21 (DE3), His- Rodβ leader-Ltnβ variant fusion peptide fractions were purified by IMAC from the cell pellet. Subsequently, the His-tag and leader peptide were cleaved off using His-AprE *in vitro*. The obtained core peptide fractions underwent purification using a C_18_ reverse-phase column and HPLC, followed by analysis through LC-MS. As shown in Figure 7B, the [M + 4H]^4+^ peptide ions observed at *m*/*z* 702.84 aligned with the 7-fold dehydration and 2-fold reduction of the Ltnβ variant. The MS/MS spectrum of the modified Ltnβ variant, illustrated in Figure 7C, revealed that only one of the first two Thr residues may undergo dehydration, resulting in one less dehydration event than anticipated, two reduction events occurring at the Dha in position 9 and Dhb in position 12 of the peptides, resulting in the formation of d-Ala and d-Abu, respectively. This sheds light on the d-Ala and d-Abu incorporation capability of RodJ_A_ and its potential for bioengineering, positioning it as a promising dehydrogenase for the ribosomal synthesis engineering of natural products containing two different d-amino acids. Nevertheless, the identification of incompletely modified Ltnβ variant peptides (one less dehydration) in this study emphasizes the importance of enhancing the modification efficiency of the rodencin modular expression system for lanthipeptide engineering.

Comparison of the amino acid sequence of RodJ_A_ with other reported LanJ_A_s revealed that RodJ_A_ shares approximately 51% sequence identity with LtnJ_A_ (SI, Figure S1B). RodJ_A_ exhibits conserved domains and residues similar to LanJ_A_-like proteins,^30^ but some unconserved residues were also identified. For instance, in RodJ_A_, the residues Arg^100^ and Asp^101^ within the conserved domain differ from the Thr/Ser and Asn found in other LanJAs. Additionally, Met^112^ and Ser^113^, located near the presumed zinc ligand (SI, Figure S1B,S1C), differ from Ile and Pro observed in other LanJAs. Further investigations through structural analysis and mutagenesis studies are necessary to pinpoint the crucial residues for RodJ_A_ function and how alterations at this site may potentially influence substrate specificity.

## Experimental Section

### General Experimental Procedures

All molecular biology experiment reagents were sourced from Thermo Fisher Scientific (Waltham, MA, USA) unless specified otherwise; all other chemicals were purchased from Sigma-Aldrich (St. Louis, MO, USA). Lipid II was synthesized and purified according to a previously published method and was generously provided by Prof. Dr. E. J. (Eefjan) Breukink.^48^

### Bacterial Strains, Plasmids, and Growth Conditions

Strains and plasmids used in this study are listed in SI, Table S2. *Bacillus subtilis* EH5 as natural producer of rodencin was used for peptide expression and molecular cloning. A single colony of *B. subtilis* EH5 was inoculated into starter liquid LB cultures and incubated overnight, then 1:100 diluted in minimal expression medium (MEM) and grown 48 h at 30 °C under shaking (220 rpm).

*Escherichia coli* TOP10 and *E. coli* BL21 (DE3) was used for peptide and protein expression. *E. coli* was grown in LB broth (Foremedium) or LB agar (Foremedium) at 37 °C, supplemented with kanamycin (100 μg/mL) and/or spectinomycin (100 μg/mL), or chloramphenicol (10 μg/mL) for plasmid selection. For peptide and protein expression, overnight cultures were 1:100 diluted in fresh LB medium and induced with IPTG (0.5 mM) at an optical density at 600 nm (OD_600_) of 0.6–0.8.

*Bacillus cereus* ATCC14579, *Bacillus subtilis* 168, *Straphylococcus aureus* LMG15975, *Straphylococcus aureus* LMG 10147, *Listeria monocytogenes* LMG10470, *Enterococcus faecium* LMG16003, *Salmonella enterica* LMG 07233, *Micrococcus flavus* NIZO B423, *Lactococcus lactis* NZ9000, and *E. coli* LMG 8223 were used as indicator strains for antimicrobial activity assays and grown in corresponding broth for preparing the overnight cultures. *B. cereus* and *S. aureus* were grown in LB broth at 37 °C under shaking (220 rpm) or LB with 1% agar (w/v), and *M. flavus* NIZO B423 was grown in LB broth at 30 °C under shaking (220 rpm) or LB with 1% agar (w/v). *E. faecium* was grown at 37 °C in brain heart infusion (BHI) broth (Sigma-Aldrich) or BHI broth containing 1% (w/v) agar. *L. monocytogenes* and *S. enterica* were grown in BHI broth at 37 °C under shaking (220 rpm) or BHI broth containing 1% (w/v) agar. *L. lactis* was grown at 30 °C in M17 broth (Oxoid) or M17 broth with 1% agar (w/v) containing 0.5% (w/v) glucose (GM17).

### Genome Mining for Novel BGCs in *B. subtilis* EH5

The draft genome sequence of *B. subtilis* EH5 was downloaded from the NCBI database (registration number RPHE01000000). The web service antiSMASH 5.0^49^ (v5.0, bacterial version) was used to identify and analyze secondary metabolite “biosynthetic gene clusters” (BGCs) in “relaxed” strictness and “All on” extra features. BGCs that have different numbers of genes or show less than 70% protein identity to the reported ones were regarded as novel. Potential novel BGCs known to encode lanthipeptides were then validated using BAGEL4.^50^ The cleavage site and ring formation of precursor peptides were predicted in RiPPMiner.

### Extraction and Purification of Rodencin from *B. subtilis* EH5

The overnight culture of *B.subtilis* EH5 was 100 times diluted in minimal expression medium (MEM) and grown for 48 h at 30 °C under shaking (220 rpm). Subsequently, the culture was centrifuged at 15 000*g* for 15 min, and the supernatant was collected and then applied to C_18_ Silica gel spherical (Sigma-Aldrich) equilibrated with 0% acetonitrile (MeCN) (VWR) containing 0.1% trifluoroacetic acid (TFA) (Sigma-Aldrich). After binding, a first wash was done with 0% MeCN containing 0.1% TFA, and then a gradient of different concentrations of MeCN containing 0.1% TFA were used to elute peptides until 100% MeCN containing 0.1% TFA. All eluted fractions were analyzed by MALDI-TOF MS to detect the target peptides. The fractions containing the target peptides were further purified on a 1260 Infinity HPLC system (Agilent) with a Aeris 3.6 μm peptide XB-C_18_ column (250 mm × 4.6 mm, 100 Å) (Phenomenex). MeCN was used as the mobile phase, and a gradient MeCN containing 0.1% TFA over 30 min at 1 mL/min was used for separation. The separated peptides were collected and lyophilized.

### Molecular Cloning

Plasmids encoding the peptides and proteins were constructed by using standard molecular cloning techniques as described previously,^51^ and all plasmid information is shown in SI, Table S2. Oligonucleotide primers used in this study were purchased from Biolegio BV (Nijmegen, The Netherlands) and are listed in SI, Table S3. All PCRs were conducted with Phusion DNA polymerase (Thermo Fisher Scientific), and the obtained PCR products were purified using a NucleoSpin Gel and PCR cleanup kit (Macherey-Nagel).

For heterologous expression of rodencin, the original genetic sequences of RodA1, RodA2, RodM1, RodM2, and RodJ_A_ and AprE were amplified directly from the *B. subtilis* EH5 genomic DNA using primers to introduce overhangs for ligation. The vectors were also PCR amplified using primers to introduce complementary overhangs. The vector and the corresponding insertion fragments were mixed and treated with Gibson Assembly Master Mix (Bioke, The Netherlands), and the mixtures were then directly applied to transform chemically competent *E. coli* TOP10 to generate plasmids. RodA1 and RodA2 were inserted downstream of T7(1) of pCDFDuet-1 to generate pCDF-His- RodA1 and pCDF-His- RodA2, respectively. The N-terminal His-tag remained from the substrate, of which the pCDF backbone was amplified. RodM1 and RodM2 were cloned into pRSFDuet-1 behind T7(1), respectively, to construct pRSF-RodM1 and pRSF-RodM2. Then RodJ was placed downstream of T7(2) in these two constructions to create pRSF-RodM1-RodJ and pRSF-RodM2-RodJ. The plasmid pACYC-His-AprE was constructed by placing the nucleotide sequence encoding (AprE) downstream of T7(1) of pACYCDuet-1.

For the construction of plasmid mutants, the template plasmid was amplified using a pair of primers designed to introduce mutations. Self-ligations of the PCR products were carried out with T4 DNA ligase (Thermo Fisher Scientific), after which competent *E. coli* TOP10 was transformed with the resulting ligation mixture. The plasmid pCDF-His-Sac and pCDF-His-Ltn were created using pCDF-His-RodA2 as template for PCR amplification introducing the mutations and 5′-phosphorylation, followed by T4 self-ligation.

Plasmid DNA was isolated from transformants using a NucleoSpin Plasmid EasyPure kit (Macherey-Nagel), and correct transformants were selected after DNA sequencing by Macrogen Europe (Amsterdam, The Netherlands). For expression purposes, chemically competent *E. coli* BL21 (DE3) was transformed with sequenced plasmid DNA from *E. coli* TOP10.

### Peptide and Protein Expression

Expression of His-RodA1 + RodM1-RodJ, His-RodA2 + RodM2-RodJ, His-RodA1 + RodM2-RodJ, His-RodA2 + RodM1-RodJ, His-RodA1 + RodM1, His-RodA2 + RodM2, His-Sac + RodM2-RodJ, and His-Ltn + RodM2-RodJ were performed according to the following protocol. For each, an overnight culture was made from several colony transformants from a plate. The overnight culture was 1:100 diluted in fresh expression culture and incubated at 37 °C under shaking (220 rpm) until an optical density at 600 nm (OD_600_) of 0.6–0.8 was achieved. The culture was then placed at 18 °C (220 rpm) with 10 min precooling and induced with 0.5 mM IPTG solution. After an overnight induction, the expression cultures were harvested.

Expression of His-AprE was done identically with that of His-RodA1 + RodM1-RodJ until induction. The expression culture was induced with 0.5 mM IPTG solution without precooling and grown for 4 h at 37 °C under shaking (220 rpm), after which the cells were harvested.

### Peptide and Protein Purification

The harvested cells were washed once in binding buffer (20 mM H_2_NaPO_4_ (Merck), 0.5 M NaCl (VWR), and 20 mM imidazole (Merck), pH 7.4), resuspended in 1/20 culture volume binding buffer, and then sonicated on ice until visible lysed. The lysate was centrifuged twice at 10 000*g*, 4 °C, for 30 min and filtered through a syringe filter (0.45 μM) to remove cell debris. His-tag purification was conducted following a standard procedure. Binding buffer (20 mM H_2_NaPO_4_, 0.5 M NaCl, and 30 mM imidazole, pH 7.4) was run over an open column containing Ni-NTA slurry (Qiagen) to equilibrate it. Then, the lysate flowed through the column twice to allow the His-peptide/protein to bind to the Ni-NTA slurry. After binding, a first wash was done with binding buffer, and a second wash was done with wash buffer (20 mM H_2_NaPO_4_, 0.5 M NaCl, 30 mM imidazole, pH 7.4), followed by elution with elution buffer (20 mM H_2_NaPO_4_, 0.5 M NaCl, 300 mM imidazole, pH 7.4). The eluted peptides and proteins were analyzed by tricine SDS-PAGE. and buffer exchange was performed using a PD-10 desalting column for subsequent leader peptide cleavage step.

### Tricine SDS-PAGE

Tricine SDS gels (16%) were prepared as described previously.^52^ Then 12 μL of His-tag elution + 3 μL of 5× loading buffer (550 mm dithiothreitol (Sigma-Aldrich), 250 mm Tris-HCl (Boom), 50% glycerol (Boom), 10% sodium dodecyl sulfate (Sigma-Aldrich), and 0.5% Coomassie Blue R-250 (Bio-Rad), pH 7.0) was boiled for 8 min, and the samples were run next to a PageRuler (Thermo Scientific) prestained ladder. After running, the gel was stained with Coomassie Brilliant Blue.

### Cleavage of Peptides with His-AprE

The buffer of His-tag-purified peptides was exchanged with 50 mM Tris-HCL buffer (pH 7.5). Then a 5 mL portion of precursor peptide solution was digested by 500 μL of AprE at 37 °C for 3 h. The cleavage of leader peptide was then observed by MALDI-TOF MS. Further purification of digested core peptides was carried out using C18 open column and RP-HPLC as described in the previous method section.

### Mass Spectrometry Analysis

A matrix-assisted laser desorption ionization-time-of-flight (MALDI-TOF) mass spectrometer analysis was performed using a 4800 Plus MALDI TOF/TOF analyzer (Applied Biosystems) in MS linear midmass positive mode. Sample preparation in brief: each sample (1 μL) was spotted on the target and dried at room temperature. Then, 0.8 μL of a 5 mg/mL α-cyano-4-hydroxycinnamic acid matrix was spotted over the samples and left to dry.

Liquid chromatography–demonstration mass spectrometry (LC–MS/MS) was performed to gain insight into the lanthionine ring pattern. LC-MS was recorded using an Ultimate 3000 UPLC system coupled with a Q-Exactive mass spectrometer, an ACQUITY BEH C_18_ column (2.1 mm × 50 mm, 1.7 μm particle size, 200 Å; Waters), a HESI ion source, and a Orbitrap detector. In each run, a 10 μL sample was injected and separated with a gradient MeCN containing 0.1% formic acid (v/v) at a flow rate of 0.5 mL/min. MS/MS data was acquired in a separate run in PRM mode selecting the doubly or triply charged ion of the targeted compound.

### NEM Reaction

The *N*-ethylmaleimide (NEM)-free cysteine assay was performed as described previously.^53,54^ An aliquot of peptides was diluted in PBS buffer, and 5 mM TCEP was mixed with 10 μL of the dissolved peptide and incubated for 30 min at room temperature. The NEM-free cysteine assay of TCEP-reduced samples was done by adding 25 mg/mL NEM. The sample was then incubated for 60 min at room temperature and used to perform MALDI-TOF MS analysis. The positive control sample is a linear peptide that has one dehydrobutyrine (Dhb) and one free cysteine with the amino acid sequence: Nisin leader-G I L G N I V G M G K Q V V Dhb G L N G L C.

### Antimicrobial Activity Assay

*B. cereus* ATCC14579, *B. subtilis* 168, *S. aureus* LMG15975, *S. aureus* LMG 10147, *L. monocytogenes* LMG10470, *E. faecium* LMG16003, *S. enterica* LMG 07233, *M. flavus* NIZO B423, *L. lactis* NZ9000, and *E. coli* LMG 8223 were used as indicator strains for antimicrobial activity assays. For each, 100 μL of overnight culture were added to 100 mL of corresponding melted agar medium. Of this mixture, 12 mL was added to each 90 mm diameter Petri dish, A sample of 8 μL of Rodα, Rodβ, and Rodβ mutant separately or combined with each other was then dropped on the plate after the agar was solid. The plates were transferred to an incubator for incubation overnight. The antimicrobial activity was determined by the presence or absence of the zones of growth inhibition. All of the tests were performed in triplicate.

MIC values were determined by broth microdilution assay.^55^ In brief, the strains were adjusted to a final concentration of 5 × 10^5^ CFU/mL in cation-adjusted Mueller–Hinton broth (MHB). After 20 h of incubation, the sample was allowed to stand at 37 °C. MIC was defined as the lowest antimicrobial concentration that did not result in visible growth. All tests were performed in triplicate.

### Liquid Growth Inhibition Assay

To test for the optimal inhibitory radio of Rodα and Rodβ, the overnight *S. aureus* culture was adjusted to a final concentration of 5 × 10^5^ CFU/mL in fresh liquid media and distributed into a 96-well plate to give a final volume of 50 μL in each well after addition of serial dilutions of Rodα and/or Rodβ. Subsequently the 96-well plate was incubated at 37 °C for 16 h, and the growth inhibition was determined by the measurement of the OD_600_. A negative control was conducted using sterile H_2_O.

To detect the sequential effects of Rodα and Rodβ on target cells, the overnight *S. aureus* culture was diluted to a final concentration of 10^8^ CFU/mL with fresh LB broth and 200 μL of diluted culture was then challenged with Rodα or Rodβ (at concentrations of 1 × MIC) in culture tubes. The tubes were incubated at 37 °C for 30 min prior to centrifugation at 10 000*g* for 2 min. The supernatants were removed from each tube, and the cell pellets were washed three times with LB broth and then resuspended in 200 μL of LB broth. Cells that had been treated with only Rodα were added to microtiter wells containing Rodβ in a 96-well plate, and cells that had been treated with only Rodβ were added to microtiter wells containing Rodα (at concentrations of 1 × MIC). The plate was incubated at 37 °C and monitored at hourly intervals for 12 h. Cells treated with Rodα and Rodβ mixed in a 1:1 ratio were used as controls. Each experiment was performed in triplicate.

### Spot on Lawn Assay

Lipid II was synthesized and purified as described in a previous study.^56^ Purified lipid II was stored in CHCl_3_/MeOH (2:1) at −20 °C until use.^44,57^ Then 100 μL of overnight *M. flavus* culture was added to 100 mL of melted MHB broth containing 1% (w/v) agar medium. Of this mixture, 12 mL was added to each 90 mm diameter Petri dish. The binding of peptide with lipid II was evaluated by spotting of lipid II (300 μm, 2.5 μL) to the edge of inhibition halo of peptides (the distance was identified by a pre-experiment). Briefly, peptide solutions were first loaded to the agar plate and left to dry, then purified lipid II was spotted to the edge of the inhibition halo of peptides or nisin (positive control). After the lipid II solution had dried, the plates were transferred to a 30 °C incubator for overnight incubation.

### Membrane Integrity Assay

The membrane integrity was detected with the membrane impermeable DNA-binding stain propidium iodide (PI, Thermo Scientific). Briefly, overnight, *M. flavus* cultures were diluted 1:100 in fresh LB broth and grown at 30 °C to an OD_600_ of 1. The cells were washed three times with PBS buffer and adjusted to an OD_600_ of 0.5, followed by the addition of 1 μM PI in the presence of peptides or nisin (positive control). The fluorescence was monitored at an excitation/emission wavelength of 535/615 nm every 5 min for 2 h at 30 °C with a Varioskan microplate reader (Thermo Scientific). All the tests were performed in triplicate.

### Assessment of Outer Membrane Permeability

The integrity of the outer membranes was analyzed with the fluorescent probe 1-*N*-phenylnaphthylamine (NPN, Sigma-Aldrich). Briefly, *E. coli* cultured overnight were diluted 100 times into fresh LB broth and grown at 37 °C to an OD_600_ of 1. The cells were washed three times with GHEPES buffer (5 mM HEPES buffer containing 5 mM glucose) and standardized to an OD_600_ of 0.5 in the GHEPES buffer. NPN was added to the cells in the presence of peptides or polymyxin B (positive control) at a final concentration of 30 μM. The fluorescence was monitored at an excitation/emission wavelength of 350/420 nm every 5 min for 2 h with a Varioskan microplate reader. All the tests were performed in triplicate.

### Protein Modeling for LanJ_A_s

The AlphaFold was employed for predicting the 3D structures of proteins.^58^ Within AlphaFold, the FASTA file encompasses the single amino acid sequence of LanJA enzymes, serving as the basis for monomer folding. The resulting model of LanJA, predicted by AlphaFold, was presented and scrutinized using the PyMOL molecular visualization system.

## Data Availability

All data supporting the findings of this study are available within the paper and its Supporting Information.

## Supporting Information Available

Structural and phylogenetic analysis of RodJ_A_; MALDI-TOF-MS mass spectra for Rodβ isolated from the supernatant of *Bacillus subtilis* EH5; tricine-SDS-PAGE analysis of the expression of rodencin; expression and activity analysis of rodencin variants lacking d-amino acids; positive control for *N*-ethylmaleimide (NEM) reaction; a table listing the minimal inhibitory concentration (MIC) of rodencin; a table summarizing the plasmids and strains used in this study; and a table summarizing the primers used in this study (PDF)
