# Supplementary material for: Discovery, Biosynthesis, and Characterization of Rodencin, a Two-Component Lanthipeptide, Harboring d-Amino Acids Introduced by the Unusual Dehydrogenase RodJA
Source: J Nat Prod. 2024 Sep 20;87(10):2344–54. doi: 10.1021/acs.jnatprod.4c00170 (PMC11519912; doi:10.1021/acs.jnatprod.4c00170)
Supplement: Supplementary file 1 — np4c00170_si_001.pdf [file np4c00170_si_001.pdf]

Supporting Information

Discovery, biosynthesis, and characterization of rodencin, a lanthipeptide incorporating D-amino acids with the unusual dehydrogenase RodJ$_A$

Yuxin Fu[1], Eleftheria Pateri[1], Oscar P. Kuipers[1]*

[1] Department of Molecular Genetics, Groningen Biomolecular Sciences and Biotechnology Institute, University of Groningen, Groningen 9747 AG, The Netherlands

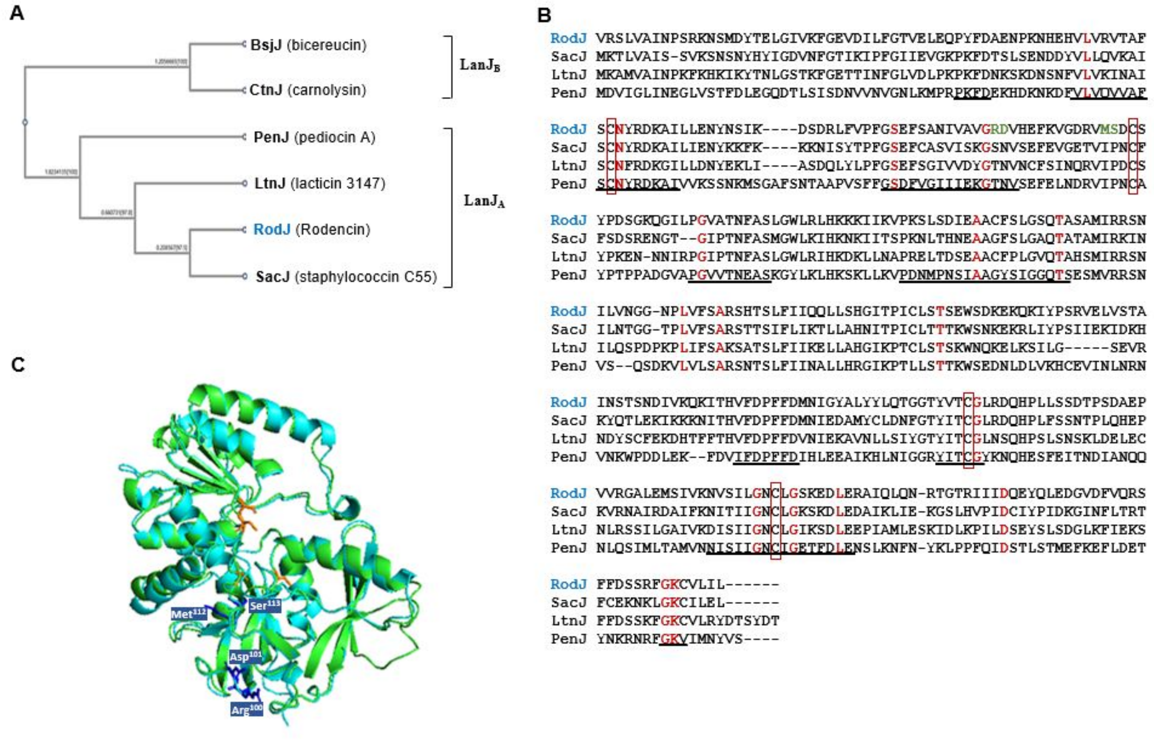

Figure S1. Structural and Phylogenetic Analysis of RodJ$_A$. (**A**) Phylogenetic tree of RodJ$_A$ and its evolutionarily related LanJs. (**B**) Alignment of the amino acid sequence of RodJ$_A$ with other reported LanJ$_A$s. Residues in red are 100% conserved, some of them have been proved to be important for LtnJ completed function; solid lines represent domains conserved in LtnJ-like proteins; Four conserved cysteines (zinc ligands) are highlighted in open boxes; Some interesting deviations (Arg^{100}, Asp^{101}, Met^{112} and Ser^{113}) were observed in RodJ$_A$ are highlighted in green. (**C**) Alignment of the 3D structures modeled for RodJ$_A$ (in green) and LtnJ$_A$ (in cyan). The four conserved cysteines, believed to act as a zinc ligand, are highlighted in orange. The non-conserved residues (Arg^{100}, Asp^{101}, Met^{112} and Ser^{113}) in RodJ$_A$ within the conserved domain or located near the presumed zinc ligand are represented in blue. The overall 3D structures were predicted using Alphafold, and the cartoons were generated with PyMol.

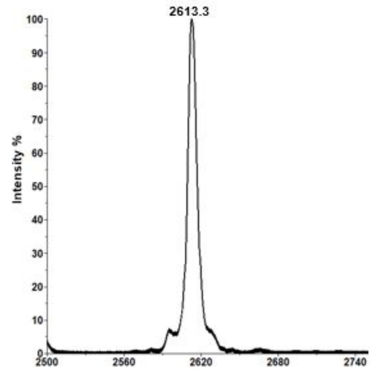

Figure S2. Maldi-Tof-MS mass spectra for Rodβ isolated from the supernatant of *Bacillus subtilis* EH5.

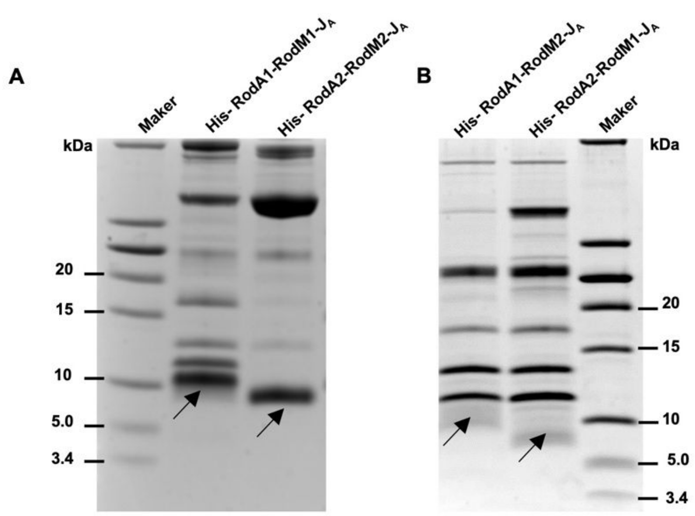

Figure S3. Tricine - SDS - PAGE analysis of the expression of Rodencin. (**A**) Tricine - SDS - PAGE analysis of the expression of RodM1-J_A modified His- RodA1 and RodM2-J_A modified His- RodA2. (**B**) Tricine - SDS - PAGE analysis of the expression of RodM2-J_A modified His-RodA1 and RodM1-J_A modified His-RodA2.

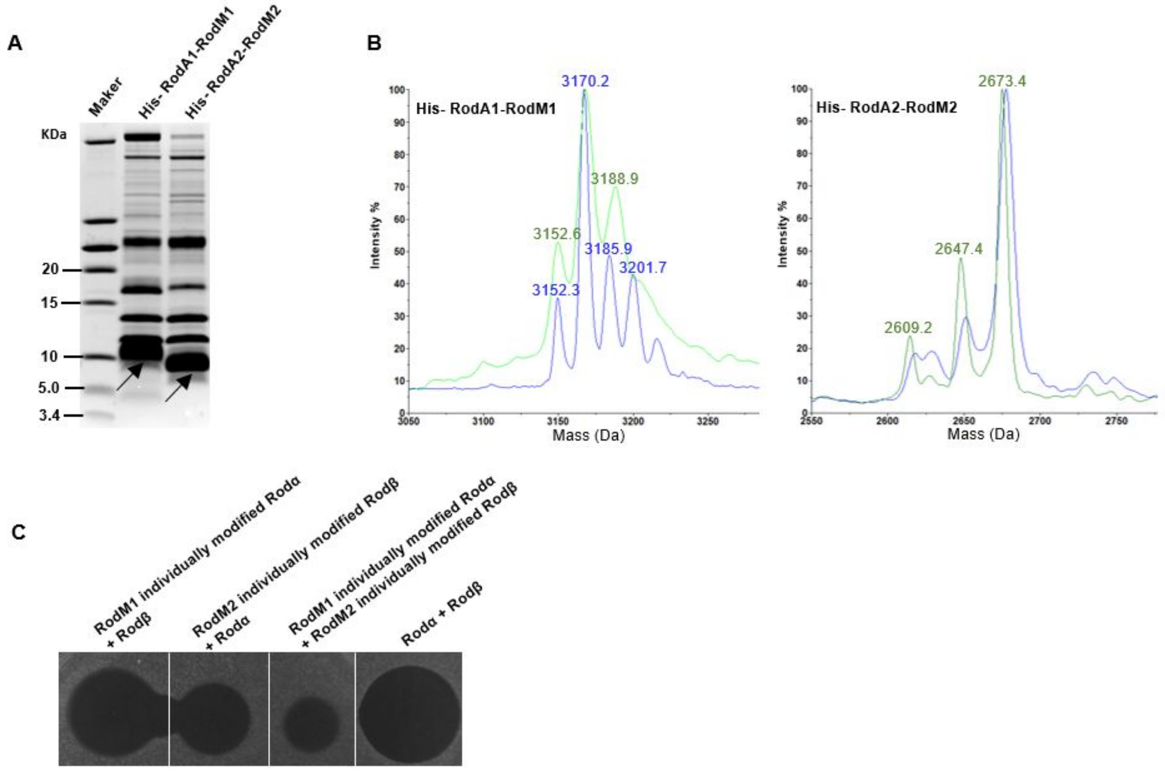

Figure S4. Expression and activity analysis of Rodencin variants lacking D-amino acids. (**A**) Tricine-SDS-PAGE analysis of the expression of RodM1-modified His-RodA1 and RodM2-modified His-RodA2. (**B**) Maldi-Tof-MS mass spectra for RodM1-modified His-RodA1 and RodM2-modified His-RodA2 before (blue) and after (green) NEM reaction. (**C**) Antimicrobial activities of Rodα and Rodβ variants lacking D-amino acids, using *M. flavus* NIZO B423 as the indicator strain. The total peptide amount spotted: 0.2 nmol.

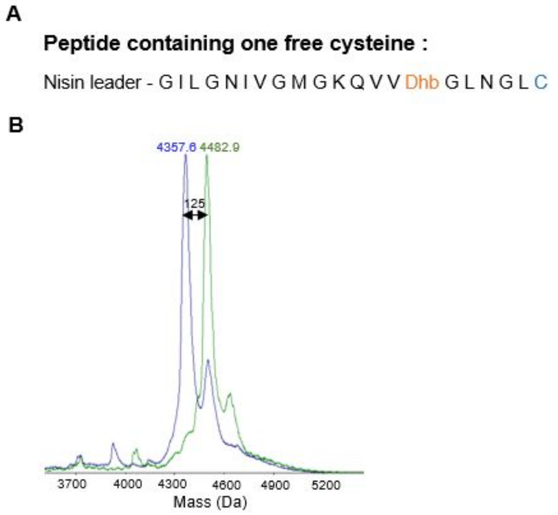

Figure S5. Positive control for N-ethylmaleimide (NEM) reaction. (**A**) The sequence of the peptide containing one free cysteine used as a positive control for the NEM reaction. (**B**) The Maldi-Tof-MS mass

spectra of peptide containing one free cysteine before (blue) and after (green) NEM reaction; the 125 Da mass shift is labeled. Related to Figure 3A and 4A.

Supplemental Table 1. Minimal inhibitory concentration (MIC) of rodencin

Strain	Rodencin (Rodα+Rodβ, ratio 1:1)<br>MIC (μM)
Gram-positive bacteria:	
Staphylococcus aureus LMG 8224	0.156
Staphylococcus aureus LMG 15975	0.625
Listeria monocytogenes LMG 10470	0.156
Bacillus cereus 14579	0.625
Bacillus subtilis 168	0.156
Enterococcus faecium LMG 16003	5
Lactococcus lactis NZ9000	0.078
Micrococcus flavus NIZO B423	0.078
Gram-negative bacteria:	
Escherichia coli LMG 8223	>20
Salmonella enterica LMG 07233	>20

Supplemental Table 2. Stains and plasmids used in this study.

Strain or plasmid	Characteristics and purpose	Source or reference
**Strains**		
Bacillus subtilis	EH5, balucin producer strain	Lab collection
Escherichia coli	TOP10, plasmids construction strain	Lab collection
Escherichia coli	BL21 (DE3)	Lab collection
Bacillus Cereus	ATCC14579, indicator strain	Lab collection
Straphylococcus aureus	LMG15975, indicator strain	Lab collection
Staphylococcus aureus	LMG 8224	Lab collection
Lactococcus lactis NZ9000	MG1363 derivative; indicator strain	Lab collection
Micrococcus flavus	NIZO B423, indicator strain	Lab collection
Enterococcus faecium	LMG16003, indicator strain	Lab collection
Listeria monocytogenes	LMG10470, indicator strain	Lab collection
Bacillus cereus 14579	indicator strain	Lab collection
Bacillus subtilis 168	indicator strain	Lab collection
Escherichia coli	LMG 8223, indicator strain	Lab collection
Salmonella enterica	LMG 07233, indicator strain	Lab collection

Plasmids		
pCDF-His-RodA1	pCDFDuet-1 derivative, expression his6-RodA1, SPE^r	This work
pCDF-His- RodA2	pCDFDuet-1 derivative, expression his6-RodA2, SPE^r	This work
pCDF-His-Rodβ leader-Ltnβ variant	pCDFDuet-1 derivative, expression his6-Ltn variant, SPE^r	This work
pRSF- RodM1	pRSFDuet-1 derivative, expression synthetase RodM1, KAN^r	This work
pRSF- RodM2	pRSFDuet-1 derivative, expression synthetase RodM2, KAN^r	This work
pRSF- RodM1-$RodJ_A$	pRSFDuet-1 derivative, expression synthetases RodM1 and $RodJ_A$, KAN^r	This work
pRSF- RodM2-$RodJ_A$	pRSFDuet-1 derivative, expression synthetases RodM2 and $RodJ_A$, KAN^r	This work
pACYC-His-AprE	pACYCDuet-1 derivative, expression His-AprE (from *B. subtilis* EH11), Cm^r	This work

Supplemental Table 3. Primers for PCRs used in this study.

Fragments	primers	Nucleic acid sequences (5' to 3')
RodA1	PN1	GCCATCATCATCATCATCACATGAAGACTGAATTCAGTAAATTACAAAAAGAGGTTAAAC
	PN2	CATTATGCGGCCGCAAGCTTTTAACACCAAGCCATACATTCTTTACTGACAGTAC
RodA2	PN3	GCCATCATCATCATCATCACATGAATGATAAAAAGTCTATTGCAAATAATAGTTTAAAAAACG
	PN4	CATTATGCGGCCGCAAGCTTTTAACAAGCGCGAGTACAAGCAGTAGTC
pCDF-His	PN5	AAGCTTGCGGCCGCATAATGCTTAAG
	PN6	GTGATGATGATGATGATGGCTGCTGCCCATG
pRSF	PN7	TGCTTAAGTCGAACAGAAAGTAATCGTATTGTAC
	PN8	GGTATATCTCCTTATTAAAGTTAAACAAAATTATTTCTACAGG
RodM1	PN9	CTTTAATAAGGAGATATACCATGGTTATATATAGGAAACAGTTTTACCCGGAACTTG
	PN10	TCTGTTCGACTTAAGCATCATCATGTTAGCGTTAAAGGGGAAATTTTAATTTTAGGATC
RodM2	PN11	CTTTAATAAGGAGATATACCATGCAATTGGGCACATAAAATAGTCCTTGCTG
	PN12	TCTGTTCGACTTAAGCATCACTAATGAACGCACTCCATATCCTCCTTATATATATTTCG
pRSF- RodM1	PN13	CTCGAGTCTGGTAAAGAAACCGCTG
pRSF- RodM1	PN14	ATGTATATCTCCTTCTTATACTTAACTAATATACTAAGATGGGGAATTG
$RodJ_A$	PN15	TATAAGAAGGAGATATACATGTGCGTTCATTAGTGGCAATTAATCCGAGTAGAAAG
	PN16	GTTTCTTTACCAGACTCGAGTTAAAGAATCAGAACACACTTTCCAAATCTGGAGGAATC

AprE	PN17	ATCATCATCATCATCACGTGAGAAGCAAAAAATTGTGGATCAGCTTGTTGTTTGC
	PN18	ATGCGGCCGCAAGCTTTTATTGTGCAGCTGCTTGTACGTTGATTAACCCTTTTC
pACYC-His	PN19	TAAAAGCTTGCGGCCGCATAATGCTTAAGTCG
	PN20	GTGATGATGATGATGATGGGTATATCTCCTTATTAAAG
Ltnβ variant	PN21	CTCCAGCGATTAGCATTCTGACTGCGTATATTTCAAGTAACACTTGTCCGACTACTGCTTGTAC
	PN22	ATGCTAATCGCTGGAGTCGCCGGAGTTGTACCACCAACAACATCATCTTCAGTTAATGCAATC
Sequencing primer		ATTTCGATTATGCGGCCGTGTACAATAC
Sequencing primer 1		ATGCGACTCCTGCATTAGGAAATTAATACG
Sequencing primer 2		ATTTCGATTATGCGGCCGTGTACAATACG
Sequencing primer 3		CAGAAAGTAATCGTATTGTACACGGCCG
Sequencing primer 4		GCCCCAAGGGGTTATGCTAGTTATTG
